# Establishing AI-driven evaluation standards and human-in-the-loop guidance for digital first aid education: a perspective

**DOI:** 10.3389/fpubh.2026.1839852

**Published:** 2026-05-21

**Authors:** Yuqing Song, Xiuxiang Luan, Xiaowei Chen

**Affiliations:** Yantaishan Hospital, East Campus, Yantai, China

**Keywords:** artificial intelligence, cardiopulmonary resuscitation, digital health education, human-in-the-loop, telemedicine

## Abstract

Short-form video platforms are now primary channels for public acquisition of medical and first-aid knowledge, including cardiopulmonary resuscitation (CPR) and the Heimlich maneuver. However, content on these platforms is mainly influenced by recommendation algorithms rather than evidence-based medical standards, resulting in the risk of erroneous procedural demonstrations that may cause severe secondary injuries. This Perspective introduces a novel digital public health paradigm: by establishing standardized digital evaluation systems and integrating computer vision (CV) with multimodal artificial intelligence (AI), short-form video platforms can transition from unidirectional “knowledge dissemination media” into bidirectional ecosystems of “intelligent assessment and professional guidance.” Specifically, this framework proposes operationalizing natural language processing (NLP) to audit instructional content against clinical guidelines, and CV to track critical kinematic thresholds (e.g., maintaining 171°–180° elbow flexion and >5 cm compression depth) to automatically filter erroneous instructions. Additionally, it presents a bifurcated dynamic response model that strictly distinguishes between routine training (utilizing real-time error correction) and unexpected emergencies (mandating “Human-in-the-Loop” telemedicine intervention). The discussion concludes with an in-depth examination of the ethical imperatives related to medical device regulation, legal liability, and algorithmic equity.

## 1 Introduction: the double-edged sword of digital health education

Short-form video platforms (e.g., TikTok, YouTube Shorts) are rapidly transforming public health education. For first aid and health knowledge, including CPR, the Heimlich maneuver, and basic sports rehabilitation, these videos leverage visual intuitiveness and broad reach to overcome the temporal and spatial limitations of established offline training, thereby enhancing public first-aid awareness. Literature reviews confirm that social media has democratized health education, expanding access for diverse populations, including underprivileged and rural communities, regardless of geographic or educational barriers. However, this broad reach also accelerates what the World Health Organization (WHO) identifies as an ‘infodemic,’ characterized by the rapid spread of both accurate and inaccurate health information (1). Clinical literature substantiates the educational efficacy of this medium; for example, controlled trials demonstrate that quality-assured, guideline-compliant public videos are as effective as traditional instructor-led screencasts in imparting foundational CPR knowledge, even among medical students (2). This underscores the medium’s scalability when content validity is rigorously maintained.

Despite its benefits, the widespread adoption of digital medical education presents significant risks. Currently, medical guidance videos are primarily influenced by the “attention economy” and recommendation algorithms, rather than strict adherence to evidence-based medical standards. The absence of peer review and professional oversight has resulted in the proliferation of flawed or critically erroneous first-aid videos. Empirical cross-platform studies confirm this misalignment: there is no significant positive correlation between a CPR video’s medical quality and its viewer interaction metrics (e.g., likes, shares). Platform algorithms tend to favor concise, high-engagement content over comprehensive, evidence-based educational videos, inadvertently spreading suboptimal and misleading information that may endanger lives (1).

This misalignment fundamentally stems from a structural conflict between ‘medical logic’ and ‘algorithmic logic’. Traditional Evidence-Based Medicine (EBM) ranks systematic reviews and randomized controlled trials at the pinnacle of reliability, viewing individual anecdotes as the least credible. Conversely, social media algorithms engineer an ‘epistemic inversion’. Driven by popularity metrics (likes, shares, and watch time), these recommendation systems function as an inverted funnel, automatically amplifying emotionally charged, sensationalized micro-narratives while structurally disadvantaging rigorous, nuanced clinical guidelines. Consequently, the rapid spread of high-engagement but medically flawed first-aid content is not a mere anomaly, but a predictable byproduct of this algorithmic ecosystem, highlighting the urgent need for machine-driven, objective standards (3). Notably, professional credentials do not ensure digital instructional accuracy. A systematic evaluation across major Chinese short-video platforms (e.g., TikTok, Bilibili, REDnote) found that while 86% of CPR videos were produced by healthcare professionals, 62% demonstrated incorrect hand placement for compressions, often relying on outdated heuristics rather than current guidelines. This indicates that human expertise can degrade when adapted for short-form media, highlighting the need for objective, machine-driven standards (4). First aid procedures are biomechanically precise; shallow compressions, incorrect rates, or improper locations during CPR or the Heimlich maneuver can result in catastrophic secondary injuries, such as rib fractures or internal organ damage. Technological advancements are required to facilitate the development of a shared digital assessment system, utilizing Computer Vision (CV) and multimodal Artificial Intelligence (AI) to transition from passive video instruction to interactive, real-time intelligent assessment and professional guidance. Crucially, this analysis establishes a fundamental taxonomic distinction between two structurally distinct use cases: (1) routine training (characterized by controlled environments, tolerance for latency, consenting users, and no immediate vital risk) and (2) real emergencies (defined by hard real-time constraints, untrained bystanders, chaotic environments, and immediate medical liability). The technological architectures, ethical stakes, and regulatory implications—particularly under the IMDRF Software as a Medical Device (SaMD) risk framework—diverge fundamentally across this continuum and must be addressed with entirely distinct governance frameworks.

## 2 Establishing digital evaluation standards

To realize intelligent first-aid guidance, the primary imperative is to translate existing paper-based medical guidelines into machine-readable and computable “digital evaluation standards.” Although organizations such as the International Liaison Committee on Resuscitation (ILCOR) and the American Heart Association (AHA) have published exhaustive first-aid guidelines, automated evaluation standards tailored for digital media and video content remain a critical gap. As digital networks become the primary source of health information, it is increasingly incumbent upon healthcare providers and public health organizations to deploy strategic, scalable tools to dispel common misconceptions and counter the rapidly spreading, inaccurate medical material on these platforms (1). Currently, evaluating user-generated first-aid content relies on manual scoring tools, which are highly subjective and entirely unscalable for the massive volume of short-form videos. This profound subjectivity severely plagues both manual and AI evaluations. A 2025 comprehensive systematic review of 137 studies assessing Large Language Models (LLMs) for health advice revealed that 65.0% of evaluations relied entirely on the subjective opinions of investigators rather than established clinical guidelines. Even among certified medical professionals, manual assessment of psychomotor skills is plagued by severe inconsistency. A recent randomized trial revealed that the inter-rater reliability (Intraclass Correlation Coefficient, ICC) among human clinical experts scoring the same CPR performances was dismal at 0.391. Furthermore, clinical studies show that manual evaluation is significantly hindered by observer bias and examiner fatigue. Automated Multimodal LLM (MLLM) evaluators have been shown to process CPR skills tests significantly faster than human experts (averaging 4.8 min versus 10 to 16 min), while eliminating the subjective waning of attention (5–8). To eliminate this hazardous human subjectivity and create an unerring digital safety framework, the design of future evaluation criteria must be based on two fundamental pillars:

### 2.1 Content compliance auditing

Platforms should introduce large language models (LLMs) based on NLP and medical knowledge graphs to automatically parse first-aid instructional videos uploaded or viewed by users. The system could automatically extract procedural steps from the video’s audio, subtitles, and keyframes, and conduct cross-modal comparisons with the latest international first-aid guidelines (e.g., AHA Guidelines). For instance, in a CPR instructional video, the system must be capable of automatically identifying and flagging whether the video omits critical golden steps (such as “first assess environmental safety,” “immediately call emergency services,” or “check patient breathing”), thereby filtering or warning against non-compliant instructional content at the source. The operational feasibility of utilizing language models to parse unstructured natural language and cross-reference it against structured medical protocols has recently been validated in real-world emergency triage. For instance, an LLM-based architecture (URGENTIAPARSE) was successfully deployed to process unstructured patient complaints and match them against the formalized FRENCH triage scale across 657 real-world emergency department encounters, achieving high predictive accuracy (F1-score of 0.900) (9). This empirical evidence robustly supports the concept that AI can effectively parse and audit user-generated first-aid narratives against established clinical guidelines. To safely operationalize this NLP auditing process and mitigate the inherent risks of algorithmic hallucination, a rigorous clinical validation pipeline is mandatory. A responsible treatment requires domain-specific fine-tuning on versioned AHA/ERC guidelines, pre-deployment clinical red-teaming protocols, and the integration of contradictory audit mechanisms. Furthermore, developers must explicitly quantify the model’s ‘critical error rate’—the frequency of outputs that, if followed, would cause direct patient harm. Standardized assessment frameworks, such as the Chatbot Assessment Reporting Tool (CHART), should be integrated early in the development cycle. Operationally, the system must employ highly constrained prompt architectures that restricting LLM outputs via function calling (e.g., structured JSON formats) and explicitly instructing the model to output an ‘unknown’ status when information is missing from the video can reduce major clinical hallucinations by up to 75% (10). Such rigorous, safety-aligned prompt engineering ensures that the AI auditor adheres strictly to factual extraction rather than generative speculation. Recent clinical validations further elevate this concept by demonstrating that Multimodal Large Language Models (MLLMs), such as GPT-4o, can directly and concurrently process audio-visual data from CPR videos. Empirical studies prove these models successfully detect critical audio cues (e.g., verbalizing the call for help) and calculate visual metrics (e.g., 100–120 compressions/min), achieving an evaluation accuracy comparable to that of senior medical examiners (7).

**Figure 1:**
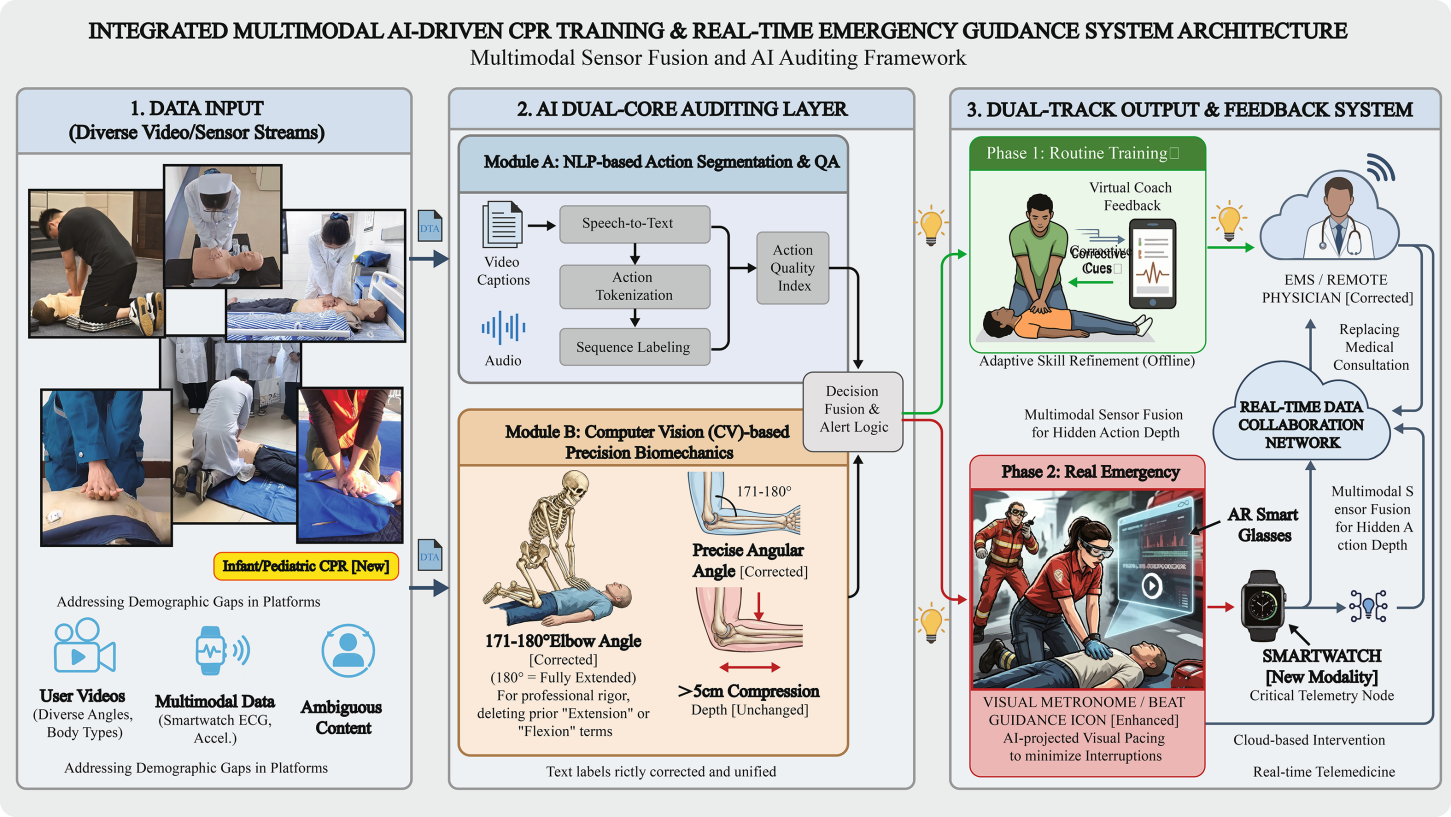
Integrated multimodal AI-driven CPR training and real-time emergency guidance system architecture.

In addition to missing steps, LLMs and temporal analysis algorithms must be deployed to detect harmful digital modifications unique to short-form media. For instance, recent studies found that 13% of CPR instructional videos artificially accelerated repetitive actions (e.g., compressions and ventilations) to fit platform time limits, severely distorting the life-saving rhythm for viewers. Furthermore, 92% omitted crucial information regarding complications and the non-absolute success rate of resuscitation (2). AI-driven temporal and content auditing can automatically flag these dangerous distortions and omissions. Integrating these LLMs allows platforms to quickly spot demographic gaps in content. For example, analyses show major platforms lack pediatric and infant CPR instructions (1). AI-driven compliance auditing can flag such critical omissions, prompting platforms to dynamically solicit and prioritize specialized content to ensure comprehensive public health coverage (Figure 1).

### 2.2 Visual and kinematic standardization

First-aid and rehabilitation operations are not merely procedural steps; they are complex biomechanical processes. Traditionally, CPR training has relied on sensor-equipped manikins that measure compression depth and frequency but fundamentally fail to monitor the rescuer’s operating posture, such as hand placement and elbow straightness. Recent biomechanical studies demonstrate that elbow flexion directly degrades compression depth and chest recoil; thus, continuous monitoring of rescuer posture through computer vision is an indispensable requirement that traditional physical sensors cannot fulfill (11).

To establish rigorous evaluation standards, we propose the prospective development of a “gold standard” 3D kinematic database. While such a comprehensive, open-access resource does not currently exist, its future construction through the collaboration of the medical community and computer scientists, utilizing high-precision motion capture of senior first-aid experts, is a necessary prerequisite for standardizing AI evaluation. Crucially, as established by the 2025 *Nature* guidelines for ethical AI benchmarking (e.g., the FHIBE dataset), constructing this database must transcend traditional non-consensual web scraping. It requires a paradigm shift toward globally diverse, explicitly consensual, and fairly compensated data collection that guarantees the right to ‘consent revocation.’ Furthermore, to protect patient and bystander privacy in chaotic emergency recordings, datasets must utilize advanced generative diffusion models to fully in-paint and replace incidental non-consensual subjects and personally identifiable information (PII). As recent studies demonstrate, traditional facial blurring remains vulnerable to re-identification, making generative in-painting an absolute prerequisite for ethical medical computer vision datasets (12). Building upon this, evaluation standards should be precisely quantified into specific visual parameters: for example, the compression rate in a CPR video demonstration must be stable at 100–120 compressions per minute, the compression depth must reach at least 5 cm, and the rescuer’s arms must remain vertical to ensure effective force transmission. Specifically, empirical trials evaluating human pose estimation in CPR reveal that maintaining an elbow angle within 171°–180° is statistically critical for achieving optimal compression depth and sufficient chest rebound. Algorithms must be calibrated to these precise angular thresholds to accurately discriminate between life-saving compressions and ineffective, energy-wasting movements (11). The clinical imperative for such precise visual calibration is not solely about efficacy, but critically about harm reduction. Data from randomized crossover trials demonstrate that CV-guided feedback systems drastically reduce the incidence of dangerously excessive compression depths—plummeting from 53.97 to 17.60% in adult cohorts—thereby systematically mitigating the risk of severe secondary injuries such as rib fractures or visceral ruptures that frequently plague unguided amateur resuscitations (13).

Beyond chest compression, kinematic standardization must encompass artificial respiration. Recent advancements utilizing Spatial–Temporal Graph Convolutional Networks (ST-GCN) have successfully quantified airway-opening efficacy, establishing that a precise “chin-frontal angle” between 40° and 49° yields optimal ventilation outcomes. The danger of relying on flawed human visual heuristics is particularly acute here. A recent randomized controlled trial demonstrated that, even under strict supervision by certified human instructors who relied on observing “visible chest rise,” trainees consistently delivered dangerously excessive tidal volumes (over 800 mL, far exceeding the AHA 500–600 mL standard). Conversely, trainees guided by an AI-integrated quantitative system consistently achieved optimal volumes. To operationalize these precise kinematic standards, pioneering open-source repositories like the “CPR-Coach” dataset have begun synchronizing RGB, optical flow, and 2D pose modalities across multiple camera perspectives to provide a rigorous training ground for advanced AI evaluators (5, 14, 15).

Recent technological breakthroughs further confirm the robustness of this approach; advanced pose-estimation algorithms like OpenPose have been successfully deployed in emergency environments to identify critical CPR posture errors—such as non-vertical compressions and unstable kneeling—even effectively overcoming visual interferences like low lighting or dark clothing. These quantified kinematic parameters will serve as the cornerstone for subsequent AI-driven determination and error correction (16). These quantified kinematic parameters form the foundation for subsequent AI-driven assessment and error correction.

## 3 AI-driven real-time evaluation and human-in-the-loop

Based on digital standards, this paper proposes a dynamic first-aid response model featuring “AI real-time calibration + telemedicine intervention.” By applying multimodal AI throughout diverse scenarios, this model aims to enhance the public’s capacity to respond to sudden emergencies.

### 3.1 Phase one: routine training—AI real-time error correction

In non-emergency scenarios, the system focuses on standardized skill training. The need for immediate, on-the-fly correction is strongly supported by recent usability studies, which reveal that learners find *post hoc* video analysis insufficient and explicitly demand real-time visual and auditory guidance (17). The pedagogical efficacy of such an autonomous “virtual coach” has now been definitively validated. A landmark 2024 RCT comparing an AI-integrated, instructor-free training system with conventional face-to-face instruction found that the AI system achieved outcomes statistically equivalent to those of conventional face-to-face instruction for chest compression accuracy and delivered significantly superior rescue breathing quality, while also boosting trainees’ psychological CPR self-efficacy (6).

Recent medical education research has shown that self-directed learning via properly curated public video platforms can effectively facilitate the acquisition of theoretical CPR knowledge (8). Building upon this strong theoretical foundation, the conceptualized AI framework presented in this Perspective introduces a psychomotor layer to bridge the gap between knowing and doing. While building upon validated CV and NLP components, this integrated architectural roadmap requires future empirical validation. By feeding “gold standard procedures” into advanced pose estimation networks, the ubiquitous smartphone is transformed into an ever-vigilant digital mentor. Users step into a virtual training crucible at home, where their front-facing camera captures and evaluates every micro-movement. To seamlessly manage these complex, multi-step procedures, the AI coach employs architectures like ST-GCN to automatically segment and classify distinct operational phases with over 87% accuracy, while continuously tracking wrist key points to ensure procedural interruptions never exceed the guideline-mandated 10-s window (16). Furthermore, this virtual coach dynamically adapts to the user’s psychological state. Emerging studies highlighted by the European Resuscitation Council (ERC) demonstrate the efficacy of deep multitask neural networks for monitoring a trainee’s real-time cognitive load, intelligently modulating scenario complexity to prevent novice rescuers from becoming overwhelmed (18).

However, the accuracy of pose-estimation algorithms is highly sensitive to the physical recording environment. Suboptimal smartphone placement can significantly impair depth and recoil estimations (17). Consequently, the AI interface should incorporate an Augmented Reality (AR) alignment guide.

To fundamentally overcome optical blind spots, this AI ecosystem must evolve into a multimodal sensor-fusion network by integrating ubiquitous consumer wearables. Recent empirical studies demonstrate that neural network models trained exclusively on noisy smartwatch accelerometer data can autonomously predict CPR compression depth with an exceptional accuracy of ±3.8 mm. Fusing kinematic visual data with wearable IoT telemetry creates a robust evaluation matrix even when the smartphone camera is entirely occluded (19). Finally, overcoming computational latency is critical. Recent optimizations in lightweight object-detection networks (e.g., customized YOLO architectures) have successfully boosted real-time inference speeds to 25 frames per second (FPS) on edge devices, ensuring zero-latency feedback during life-saving scenarios (16). Prototyping on embedded edge-computing platforms (e.g., Jetson Nano) proves that critical visual detection and text-to-speech logic can be executed entirely locally, ensuring fail-safe offline operation in remote or disaster-stricken environments (20).

The viability of utilizing AI as an autonomous virtual coach is now backed by empirical clinical evidence. Recent cross-sectional studies confirm that state-of-the-art MLLMs can autonomously grade complex psychomotor tasks with a reliability score of 4.17 out of 5.00 when evaluated by certified AHA instructors, proving their readiness for real-life deployment (7). Crucially, in real-world emergencies, rescuers rarely make isolated mistakes; they frequently exhibit complex ‘composite errors’ (e.g., simultaneously bending arms, applying insufficient pressure, and maintaining slow frequency). Recent advancements demonstrate that AI can overcome this exponential complexity through human-cognition-inspired architectures, enabling the virtual coach to accurately identify unseen multi-error combinations in real-time, even when trained exclusively on single-error data (15).

### 3.2 Phase two: unexpected emergencies”human-in-the-loop” telemedicine

In genuine emergencies (e.g., a sudden cardiac arrest), relying solely on AI for fully automated guidance poses extreme clinical risks. Furthermore, commercial AI models often operate under strict liability constraints that may limit their autonomous deployment in life-or-death scenarios; without specific emergency-mode fine-tuning, such models risk prioritizing generic medical disclaimers over actionable life-saving instructions. Furthermore, while MLLMs demonstrate high grading accuracy in controlled settings, their critical error rates. The frequency of outputs that would cause direct patient harm if followed—remains insufficiently audited for unsupervised deployment. Such algorithmic abstention would be fatal, reinforcing why AI cannot act as a solitary medical authority (21). This HITL principle mirrors validated architectures already deployed in emergency triage, where AI-assisted NLP parsing has been shown to effectively support—rather than supplant—clinical decision-making by nursing and physician teams in real-world emergency department workflows (9), demonstrating both the operational feasibility and the safety ceiling of such hybrid systems.

The absolute necessity of transitioning from traditional audio-only dispatcher assistance (T-CPR) to visual, “Human-in-the-Loop” telemedicine is overwhelmingly supported by evidence. An RCT published in *Scientific Reports* demonstrated that video-assisted CPR (V-CPR) soared correct hand positioning to 96.0%, compared to a meager 57.1% for audio instructions alone (21). Moreover, a 2025 RCT demonstrated that instantaneous, interactive digital guidance empowers completely untrained laypersons to achieve resuscitation metrics that eclipse those of even BLS-certified individuals acting without digital support. By mitigating the psychological barrier of ‘fear of doing harm,’ a well-designed digital interface can instantaneously bridge the fatal gap between zero medical training and effective life-saving action. In this proposed architecture, upon triggering the emergency mode, the AI would immediately assume the role of a digital vanguard (22). Slicing through this cognitive paralysis, the AI would be designed to bypass convoluted tutorials and instantly project a high-contrast, pulsating visual metronome, coupled with minimalist, rhythmic voice prompts, to minimize “hands-off” time. Simultaneously, the backend would automatically connect to Emergency Medical Services (EMS). However, because remote dispatchers struggle to visually judge physical compression depth via raw, 2D video feeds alone, an AI-augmented interface is strictly required (23). The physician monitors the live, chaotic scene while the AI projects critical auxiliary analytical data directly onto the terminal (e.g., calculating compression depth with an error of < 1 cm), turning the smartphone into a life-saving telemetry node (16).

In chaotic scenarios where optimal camera positioning is not possible, the rescuer’s smartwatch serves as a continuous telemetry node, streaming quantitative parameters directly to the EMS dashboard (19). Concurrently, the AI’s NLP capabilities can analyze the auditory environment to identify “ineffective communication” or panic among bystanders, empowering the remote physician to issue authoritative commands to stabilize the psychological state (18). Utilizing these AI-enhanced objective metrics, the physician provides indispensable, emotionally stabilizing guidance to the on-site rescuer via voice. The practical feasibility of such remote interventions is strongly supported by recent telemedicine reviews, which document multiple life-saving precedents where off-site experts successfully guided on-site personnel using standard smartphone video applications (1). This “Human-in-the-Loop” design leverages the computational advantages of AI while firmly upholding the medical decision-making baseline set by human physicians, particularly crucial given that a substantial proportion of first-aid videos circulating online are uploaded by non-professionals, thereby contributing to suboptimal procedures (2, 4).

While smartphone telemedicine provides a foundational bridge, having a rescuer juggle a handheld device while attempting strenuous bimanual chest compressions creates a severe physical bottleneck. Wearable technologies like smart glasses overcome this limitation, providing a fully hands-free resuscitation environment. Wearable AR eliminates the cognitive friction of constantly shifting gaze between a tiny screen and a dying patient. This hardware transition creates a seamless “shared vision,” enabling the remote expert to dynamically project critical holographic overlays—such as a glowing target on the patient’s sternum for hand placement or an animated defibrillator diagram—directly into the rescuer’s field of view (24). Comparative trials utilizing the Simulation Task Load Index (SIM-TLX) reveal that this spatial computing approach significantly reduces instruction time and mitigates the psychological workload of both the terrified rescuer and the remote expert (25).

## 4 Challenges and ethical imperatives

As a disruptive digital public health architecture, the implementation of this ecosystem inevitably requires overcoming a series of practical obstacles and ethical challenges:

### 4.1 Regulatory oversight

When short-form video platforms or affiliated AI software begin to provide diagnostic-natured feedback or real-time correction of first-aid actions, they cross the traditional boundary of “informational science popularization.” Regulatory bodies (such as the US FDA or the Chinese NMPA) need to determine whether such systems should be classified as “Software as a Medical Device” (SaMD). The regulatory pathway diverges fundamentally based on the use case. According to the IMDRF framework, the risk categorization of Software as a Medical Device (SaMD) is determined by the state of the healthcare situation and the significance of the information provided to the healthcare decision. An out-of-hospital cardiac arrest constitutes a ‘critical situation’. In this high-stakes environment, if an AI system’s real-time kinematic corrections are intended to ‘drive clinical management’ (i.e., guide an untrained layperson’s next intervention), the software almost certainly falls into Category III (High Impact) or Category IV (Very High Impact) if deemed to directly treat. Furthermore, under the EU AI Act (2024), such real-time interventional systems explicitly qualify as high-risk AI systems under Annex III, triggering conformity assessment obligations that go well beyond standard SaMD regulation. In stark contrast, systems utilized exclusively for routine simulation training operate in ‘non-serious situations’ and primarily ‘inform’ clinical management, placing them in the significantly lower-risk Category I or II (3, 5, 10, 18, 26).

### 4.2 Legal liability

In emergency first-aid scenarios, if the AI evaluation system fails to promptly correct a rescuer’s erroneous CPR actions due to suboptimal smartphone camera angles, poor lighting, or visual occlusions—ultimately resulting in patient death or secondary injury—how should legal liability be defined? A clear framework delineating the responsibilities of platform providers, algorithm developers, and on-site rescuers is urgently needed from the legal community. This is essential to prevent technology providers from abandoning research and development of public health technologies out of fear of litigation.

Current legislative shifts provide a preliminary analytical framework. Under the EU AI Act and the revised Product Liability Directive (2024/2853), a real-time medical guidance system causing patient harm through incorrect instructions could engage the provider’s liability regardless of generic disclaimers. Consequently, developers and regulators must navigate three competing liability models: (1) product liability (falling on the AI algorithm provider), (2) professional liability (falling on the supervising telemedicine physician), and (3) platform safe harbor limitations. This is not merely a legal nicety; it directly determines whether developers will build these systems at all. The implementation of a Human-in-the-Loop (HITL) architecture critically modifies this allocation. By retaining a licensed physician as the final medical authority, this hybrid model potentially shields technology providers from direct medical malpractice claims—shifting the locus of acute clinical liability to the human supervisor—while ensuring maximum patient safety.

### 4.3 Algorithmic bias

Pose recognition algorithms must undergo generalization validation across varying body types, skin tones, and clothing styles. A critical, unacknowledged epistemic limitation in current computer vision resuscitation research is dataset geographic bias. Many studies establishing kinematic thresholds (e.g., 171°-180° elbow angles or 40°-49° chin-frontal angles) originate predominantly from Asian research groups (11, 13, 16, 17). This raises legitimate questions about whether these thresholds generalize across morphologically diverse populations; rigorous external validation on non-Asian cohorts is urgently required. Furthermore, while initial studies show promise, robust mitigation strategies for camera-condition degradation (e.g., suboptimal lighting and extreme angles) remain insufficient. To address anthropometric variations, we propose the prospective adoption of ‘scale-invariant metrics’—normalizing spatial measurements by intrinsic body dimensions, such as shoulder width. However, it must be clearly flagged that the implementation of such scale-free representations for zero-shot generalization in CPR contexts requires extensive future empirical validation. Beyond algorithmic adjustments, the foundational training data must be radically reformed. A 2025 landmark study published in *Nature* utilizing the Fair Human-Centric Image Benchmark (FHIBE) empirically demonstrated that current pose estimation and person detection models suffer from severe ‘intersectional biases’—performing significantly worse on older individuals, darker skin tones, and underrepresented ancestries due to the non-consensual, geographically skewed nature of legacy web-scraped datasets. Therefore, achieving true algorithmic equity in resuscitation requires training CV models on consensually sourced, globally diverse datasets with self-reported demographic annotations, rather than relying on flawed, stereotyped legacy data. The urgency of this reform is amplified by recent *Nature* findings demonstrating that CV models suffer from profound ‘intersectional biases’—failing disproportionately on combinations of older age (60 + years), darker skin tones, and African ancestry. Alarmingly, direct error modeling reveals that this algorithmic degradation is heavily exacerbated by body occlusion (i.e., a low number of visible keypoints) (12). Because CPR inherently involves massive visual occlusion—with rescuer hands overlapping the patient’s chest—and is most frequently required for older adults, deploying current CV models without addressing these intersectional and occlusion-based blind spots poses an unacceptable, lethal risk to marginalized demographics.

Transitioning from controlled environments to chaotic real-world deployments also requires exceptional algorithmic resilience. A 2025 ERC scoping review revealed that current CV applications are overwhelmingly confined to static cameras in highly controlled settings (18). While initial studies show promise, robust mitigation strategies for camera-condition degradation (e.g., suboptimal lighting and extreme angles) are still in their infancy, requiring massive datasets capturing real-world environments before clinical deployment. To guarantee resilience, cutting-edge systems now employ “arm length discrimination” vectors and foreground-background modeling to mathematically compensate for erratic camera angles, perspective distortion, and environmental clutter (13). Moreover, skeleton-based pose estimation exhibits significantly superior generalization compared to traditional RGB-based visual methods in unpredictable settings (15, 16). This resilience must additionally extend to wearable sensor integration; because raw smartwatch data is intrinsically corrupted by sensor white noise and erratic hand vibrations, neural networks must be rigorously trained on highly noisy, uncurated real-world datasets to prevent hallucinatory guidance during high-stress resuscitations (19).

Beyond physical and environmental biases, algorithmic equity must encompass cognitive accessibility. AI instructional texts consistently require a college-level reading proficiency, severely violating the 6th-grade standard recommended for public patient education materials (21). To ensure equitable public health impact, NLP modules must be explicitly constrained to output layperson-friendly instructions, preventing cognitive overload for untrained bystanders during emergencies (27).

## 5 Future directions and conclusion

The era of short-form video has brought unprecedented opportunities to popularize public first-aid knowledge; however, the solemnity of medicine demands that we not merely stop at metrics like traffic and “likes.” The AI-driven evaluation standards and “Human-in-the-Loop” guidance mechanism proposed in this paper provide a viable technological pathway for addressing quality control and emergency applications in the popularization of digital first-aid science. To facilitate this paradigm shift safely, the academic and regulatory communities must implement standardized assessment frameworks—such as the emerging Chatbot Assessment Reporting Tool (CHART) to ensure that the clinical validation of these generative AI models is transparent, reproducible, and methodologically rigorous (5).

Beyond CPR and airway obstructions, this AI-driven architectural paradigm is highly scalable to other complex trauma scenarios. State-of-the-art vision models have recently been successfully trained on domain-specific datasets to autonomously triage dynamic injuries in real time—such as classifying burn severity (first to third degree) or detecting severe hemorrhages—and instantly trigger appropriate multimodal first-aid protocols (e.g., prompting direct pressure for open wounds). This multi-scenario adaptability highlights the vast potential of computer vision in comprehensive pre-hospital care (20). Recent developmental trials have successfully validated the implementation of such systems via ubiquitous smartphones—utilizing models like YOLOv8 for keypoint detection—by successfully democratizing regular training. By eliminating the need for expensive, specialized hardware (e.g., wearable accelerometers or sensor-equipped manikins), this vision drastically lowers the barrier to entry for the public (17). Moreover, the deployment of AR-driven telemedicine is becoming increasingly economically viable for bridging severe public health disparities. Contemporary research indicates that modern smart glasses are now commercially accessible at prices lower than those of high-end smartphones. By requiring only standard Wi-Fi connectivity and a video-call application, this wearable technology can democratize Basic Life Support (BLS) training and emergency teleassistance for isolated, rural populations characterized by low population density and a profound lack of on-site resuscitation instructors (24). Because these advanced networks (e.g., OpenPose) solely require a standard monocular RGB camera to process complex 2D spatial coordinates into five comprehensive biomechanical metrics—compression depth, frequency, position, chest rebound, and elbow straightness—with over 94% accuracy, any individual with a smartphone can now access clinic-grade postural assessments at zero marginal cost (11).

To address the practical challenge of hardware availability during sudden out-of-hospital cardiac arrests, future public health infrastructure should pioneer a co-location deployment strategy. By integrating lightweight, 5G-enabled AR headsets directly alongside Automated External Defibrillators (AEDs) within urban public access cabinets, bystanders can instantly initiate a hands-free, AI-assisted telemedicine connection the moment the emergency equipment is accessed (24). Taking this infrastructure integration a step further, recent clinical trials have successfully validated the next-generation of ‘Smart AEDs’ (e.g., the HeartSave M650), which directly embed Neural Processing Units (NPUs), 360-degree cameras, and wireless communication modules into the defibrillator chassis. In randomized crossover trials, these CV-integrated AEDs more than doubled the accuracy of optimal-depth compressions (from 29.54 to 60.38% in adults) while simultaneously streaming AI-derived metrics and live video to cloud-based physician terminals. This hardware evolution demonstrates that the ‘Human-in-the-Loop’ telemedicine framework can be seamlessly integrated into the very life-saving equipment bystanders instinctively reach for (13).

Achieving this vision requires close interdisciplinary collaboration among emergency medicine specialists, rehabilitation physicians, computer scientists (AI/CV developers), and short-form video platform providers. In the future, digital technology should serve not only as a conduit for knowledge dissemination but also as “Digital Guardrails” that safeguard human lives. The transition from static, unidirectional video instruction to dynamic, interactive AI evaluation and first-aid response systems represents both the trajectory of technological evolution and an inevitable trend in digital public health development.

## Data Availability

The original contributions presented in the study are included in the article/supplementary material, further inquiries can be directed to the corresponding author/s.
